# Supplementary material for: Iterative improvement in the automatic modular design of robot swarms
Source: PeerJ Comput Sci. 2020 Dec 7;6:e322. doi: 10.7717/peerj-cs.322 (PMC7924708; doi:10.7717/peerj-cs.322)
Supplement: Supplemental Information 3 [file peerj-cs-06-322-s003.zip › argos3/doc/api/standalone/a00656_source.html]

ARGoS: plugins/simulator/visualizations/qt-opengl/qtopengl\_render.cpp Source File


- Main Page
- Related Pages
- Namespaces
- Classes
- Files

- File List
- File Members

# plugins/simulator/visualizations/qt-opengl/qtopengl\_render.cpp

Go to the documentation of this file.

```
00001 
00007 #include "qtopengl_render.h"
00008 #include "qtopengl_application.h"
00009 
00010 #include <argos3/core/utility/logging/argos_log.h>
00011 #include <argos3/core/utility/string_utilities.h>
00012 #include <cstring>
00013 
00014 #include <QPixmap>
00015 #include <QSplashScreen>
00016 
00017 namespace argos {
00018 
00019    /****************************************/
00020    /****************************************/
00021 
00022    void CQTOpenGLRender::Init(TConfigurationNode& t_tree) {
00023       /* Parse options from the XML */
00024 #ifdef ARGOS_WITH_LUA
00025       GetNodeAttributeOrDefault(t_tree, "lua_editor", m_bLuaEditor, m_bLuaEditor);
00026 #endif
00027       /* Save the configuration for later */
00028       m_tConfTree = t_tree;
00029       /* Set up dummy arguments for QApplication */
00030       m_nOptionNum = 1;
00031       m_ppcOptions = new char*[m_nOptionNum];
00032       m_ppcOptions[0] = new char[7];
00033       ::strcpy(m_ppcOptions[0], "argos3");
00034       /* Create the QT application */
00035       m_pcApplication = new CQTOpenGLApplication(m_nOptionNum, m_ppcOptions);
00036       /* Set some data about the application */
00037       m_pcApplication->setApplicationName("ARGoS");
00038       m_pcApplication->setApplicationVersion("3.0");
00039       m_pcApplication->setOrganizationName("ARGoS");
00040       m_pcApplication->setOrganizationDomain("argos-sim.info");
00041       /* Draw the main window */
00042       m_pcMainWindow = new CQTOpenGLMainWindow(m_tConfTree);
00043 #ifdef ARGOS_WITH_LUA
00044       /* Create Lua editor if required */
00045       if(m_bLuaEditor) {
00046          m_pcQTOpenGLLuaMainWindow = new CQTOpenGLLuaMainWindow(m_pcMainWindow);
00047       }
00048 #endif
00049       LOG.Flush();
00050       LOGERR.Flush();
00051    }
00052 
00053    /****************************************/
00054    /****************************************/
00055 
00056    void CQTOpenGLRender::Execute() {
00057       try {
00058          /* This effectively starts the experiment */
00059          m_pcMainWindow->show();
00060 #ifdef ARGOS_WITH_LUA
00061          if(m_bLuaEditor) {
00062             m_pcQTOpenGLLuaMainWindow->show();
00063          }
00064 #endif
00065          m_pcApplication->exec();
00066       }
00067       catch(CARGoSException& ex) {
00068          THROW_ARGOSEXCEPTION_NESTED("Error while executing the experiment.", ex);
00069       }
00070    }
00071 
00072    /****************************************/
00073    /****************************************/
00074 
00075    void CQTOpenGLRender::Destroy() {
00076 #ifdef ARGOS_WITH_LUA
00077       /* Destroy the Lua editor */
00078       if(m_bLuaEditor) {
00079          delete m_pcQTOpenGLLuaMainWindow;
00080       }
00081 #endif
00082       /* Destroy the main window */
00083       delete m_pcMainWindow;
00084       /* Destroy the QT application */
00085       delete m_pcApplication;
00086       /* Get rid of the factory */
00087       CFactory<CQTOpenGLUserFunctions>::Destroy();
00088    }
00089 
00090    /****************************************/
00091    /****************************************/
00092 
00093    CQTOpenGLMainWindow& CQTOpenGLRender::GetMainWindow() {
00094       if(m_pcMainWindow == NULL) {
00095          THROW_ARGOSEXCEPTION("CQTOpenGLRender::GetMainWindow(): no main window created");
00096       }
00097       return *m_pcMainWindow;
00098    }
00099 
00100    /****************************************/
00101    /****************************************/
00102 
00103 #ifdef ARGOS_WITH_LUA
00104    CQTOpenGLLuaMainWindow& CQTOpenGLRender::GetLuaMainWindow() {
00105       if(m_pcQTOpenGLLuaMainWindow == NULL) {
00106          THROW_ARGOSEXCEPTION("CQTOpenGLRender::GetLuaMainWindow(): no Lua main window created");
00107       }
00108       return *m_pcQTOpenGLLuaMainWindow;
00109    }
00110 #endif
00111 
00112    /****************************************/
00113    /****************************************/
00114 
00115    REGISTER_VISUALIZATION(CQTOpenGLRender,
00116                           "qt-opengl",
00117                           "Carlo Pinciroli [ilpincy@gmail.com]",
00118                           "1.0",
00119                           "An interactive graphical renderer based on QT and OpenGL.",
00120                           "The QT-OpenGL renderer is a graphical renderer based on QT >= 4.5 and OpenGL.\n"
00121                           "It allows the user to watch and modify the simulation as it's running in an\n"
00122                           "intuitive way.\n\n"
00123                           "REQUIRED XML CONFIGURATION\n\n"
00124                           "  <visualization>\n"
00125                           "    <qt-opengl />\n"
00126                           "  </visualization>\n\n"
00127                           "OPTIONAL XML CONFIGURATION\n\n"
00128                           "You can auto-play the simulation at startup by specifying the 'autoplay'\n"
00129                           "attribute as follows:\n\n"
00130                           "  <visualization>\n"
00131                           "    <qt-opengl autoplay=\"true\" />\n"
00132                           "  </visualization>\n\n"
00133                           "It is also possible to set some camera parameters. There are 10 available\n"
00134                           "cameras to use. You can switch from one to the other by clicking on the\n"
00135                           "graphical view (to give it focus) and then pressing the keys 0-9.\n"
00136                           "To configure position and orientation of specific cameras, say cameras 0 to 3,\n"
00137                           "you have to include the following XML code:\n\n"
00138                           "  <visualization>\n"
00139                           "    <qt-opengl>\n"
00140                           "      <camera>\n"
00141                           "        <placement idx=\"0\" position=\"2,2,2\" look_at=\"1,1,1\" />\n"
00142                           "        <placement idx=\"1\" position=\"1,0,7\" look_at=\"1,0,0\" />\n"
00143                           "        <placement idx=\"2\" position=\"3,3,4\" look_at=\"1,6,0\" />\n"
00144                           "        <placement idx=\"3\" position=\"2,3,2\" look_at=\"0,1,0\" />\n"
00145                           "      </camera>\n"
00146                           "    </qt-opengl>\n"
00147                           "  </visualization>\n\n"
00148                           "The 'idx' attribute specifies the camera index (and the key to press to switch\n"
00149                           "to that camera).\n"
00150                           "The 'position' attribute contains the position of the camera in the arena.\n"
00151                           "The 'look_at' attribute sets the point the camera is looking at.\n"
00152                           "Sometimes, specifying a camera positioning with only 'position' and 'look_at'\n"
00153                           "generates ambiguous configurations, which ARGoS resolves in a default way after\n"
00154                           "printing a warning message. To place the camera without ambiguities, specify\n"
00155                           "also the 'up' vector of the camera. If the camera is your head, imagine this\n"
00156                           "vector as an arrow that stems from the center of your head and extends upwards\n."
00157                           "The 'up' vector must be perpendicular to the difference between the 'look_at'\n"
00158                           "and the 'position' vectors."
00159                           "You can set some optical parameters of real cameras, namely the focal length\n"
00160                           "and the length of the frame diagonal. For example:\n\n"
00161                           "  <visualization>\n"
00162                           "    <qt-opengl>\n"
00163                           "      <camera>\n"
00164                           "        ...\n"
00165                           "        <placement idx=\"4\"\n"
00166                           "                   position=\"4,1,4\"\n"
00167                           "                   look_at=\"2,1,0\"\n"
00168                           "                   lens_focal_length=\"50\"\n"
00169                           "                   frame_diagonal=\"40\" />\n"
00170                           "        ...\n"
00171                           "      </camera>\n"
00172                           "    </qt-opengl>\n"
00173                           "  </visualization>\n\n"
00174                           "The 'lens_focal_length' attribute controls the focal length of the lens of the\n"
00175                           "simulated camera. The value is in millimeters and it defaults, if not set in\n"
00176                           "XML, to 20mm.\n"
00177                           "The 'frame_diagonal' attribute specifies the length of the frame diagonal of\n"
00178                           "the image film. The value is in millimeters and it defaults, if not set in\n"
00179                           "XML, to 35mm.\n"
00180                           "This visualization also allows for user customization. In a similar fashion to\n"
00181                           "the loop functions, you can set a plug-in that derives from the\n"
00182                           "CQTOpenGLUserFunctions class. To load it in the system, follow this example:\n\n"
00183                           "  <visualization>\n"
00184                           "    <qt-opengl>\n"
00185                           "      <user_functions library=\"/path/to/libmyuserfunctions.so\"\n"
00186                           "                      label=\"my_user_functions\" />\n"
00187                           "    </qt-opengl>\n"
00188                           "  </visualization>\n\n"
00189                           "The 'library' attribute points to the library where the user functions are\n"
00190                           "stored. This library can be the same as the loop functions, or a new one.\n"
00191                           "There is no limitation to where the code is to be found.\n"
00192                           "The 'label' attribute identifies the user function class to use. In this way,\n"
00193                           "in a single library you can have multiple user function implementations, if\n"
00194                           "you wish.\n"
00195                           "You can also grab frames and store them into image files, for example to create\n"
00196                           "videos in a fast way. To do it, you just need to press the red capture button\n"
00197                           "and frame grabbing will be on. By default, the frames are named\n"
00198                           "'frame_NNNNN.png' and are stored in the current directory, i.e. the directory\n"
00199                           "where you run the 'argos' command. If you want to override this behavior, you\n"
00200                           "can add the optional 'frame_grabbing' section as follows:\n\n"
00201                           "  <visualization>\n"
00202                           "    <qt-opengl>\n"
00203                           "      <frame_grabbing directory=\"frames\"\n"
00204                           "                      base_name=\"myframe_\"\n"
00205                           "                      format=\"png\"\n"
00206                           "                      quality=\"100\" />\n"
00207                           "    </qt-opengl>\n"
00208                           "  </visualization>\n\n"
00209                           "All the attributes in this section are optional. If you don't specify one of\n"
00210                           "them, the default is taken.\n"
00211                           "The 'directory' attribute stores the directory where the frames are saved. If\n"
00212                           "the directory does not exist, a fatal error occurs. The directory must exist\n"
00213                           "and be writable. Both absolute and relative paths are allowed. The default\n"
00214                           "value is '.'\n"
00215                           "The 'base_name' attribute is the string to prepend to the file name. After this\n"
00216                           "string, the frame number (padded to 5 digits) is added. The default value is\n"
00217                           "'frame_', so a typical resulting name is 'frame_00165'.\n"
00218                           "The 'format' attribute specifies the format. The default value is 'png' but you\n"
00219                           "can put any format supported by Qt>=4.5. Refer to the Qt documentation for the\n"
00220                           "complete list of supported formats.\n"
00221                           "The 'quality' attribute dictates the quality of the image. Its value is in the\n"
00222                           "range [0:100] where 0 means maximum compression and minimum quality, and 100\n"
00223                           "means maximum quality and no compression at all. The default value is '-1',\n"
00224                           "which means to use Qt's default quality. For videos, it's best to use 100 to\n"
00225                           "avoid artifacts due to compression. For a normal screenshot, the default is the\n"
00226                           "safest choice.\n",
00227                           "Usable"
00228       );
00229 
00230 }
```

---

Generated on 10 Jul 2018 for ARGoS by 
 1.6.1
